# *In Silico* Structure-Based Repositioning of Approved Drugs for Spike Glycoprotein S2 Domain Fusion Peptide of SARS-CoV-2: Rationale from Molecular Dynamics and Binding Free Energy Calculations

**DOI:** 10.1128/mSystems.00382-20

**Published:** 2020-09-22

**Authors:** Nishant Shekhar, Phulen Sarma, Manisha Prajapat, Pramod Avti, Hardeep Kaur, Anupam Raja, Harvinder Singh, Anusuya Bhattacharya, Saurabh Sharma, Subodh Kumar, Ajay Prakash, Bikash Medhi

**Affiliations:** a Department of Pharmacology, PGIMER, Chandigarh, India; b Department of Biophysics, PGIMER, Chandigarh, India; c Department of Ophthalmology, Government Medical College & Hospital, Chandigarh, India; Princeton University

**Keywords:** severe acute respiratory syndrome coronavirus 2, fusion peptide, molecular dynamics simulation, molecular mechanics/generalized Born model and solvent accessibility, S2 fusion peptide-containing domain, coronavirus, docking, drug discovery, free energy, fusion peptide, molecular dynamics, repurposing, repositioning, spike protein

## Abstract

The membrane-anchored spike (S) protein of severe acute respiratory syndrome coronavirus 2 (SARS-CoV-2) has a pivotal role in directing the fusion of the virus particle mediated by the host cell receptor angiotensin-converting enzyme 2 (ACE-2). The fusion peptide region of the S protein S2 domain provides SARS-CoV-2 with the biological machinery needed for direct fusion to the host lipid membrane. In our present study, computer-aided drug design strategies were used for the identification of FDA-approved small molecules using the optimal structure of the S2 domain, which exhibits optimal interaction ratios, structural features, and energy variables, which were evaluated based on their performances in molecular docking, molecular dynamics simulations, molecular mechanics/generalized Born model and solvent accessibility binding free energy calculations of molecular dynamics trajectories, and statistical inferences. Among the 2,625 FDA-approved small molecules, chloramphenicol succinate, imipenem, and imidurea turned out to be the molecules that bound the best at the fusion peptide hydrophobic pocket. The principal interactions of the selected molecules suggest that the potential binding site at the fusion peptide region is centralized amid the Lys790, Thr791, Lys795, Asp808, and Gln872 residues.

**IMPORTANCE** The present study provides the structural identification of the viable binding residues of the SARS-CoV-2 S2 fusion peptide region, which holds prime importance in the virus’s host cell fusion and entry mechanism. The classical molecular mechanics simulations were set on values that mimic physiological standards for a good approximation of the dynamic behavior of selected drugs in biological systems. The drug molecules screened and analyzed here have relevant antiviral properties, which are reported here and which might hint toward their utilization in the coronavirus disease 2019 (COVID-19) pandemic owing to their attributes of binding to the fusion protein binding region shown in this study.

## Abstract (precis)

The present study provides the structural identification of the viable binding residues of the SARS-CoV-2 S2 fusion peptide region, which holds prime importance in the virus’s host cell fusion and entry mechanism. The classical molecular mechanics simulations were set on values that mimic physiological standards for a good approximation of the dynamic behavior of selected drugs in biological systems. The drug molecules screened and analyzed here have relevant antiviral properties, which are reported here and which might hint toward their utilization in the coronavirus disease 2019 (COVID-19) pandemic owing to their attributes of binding to the fusion protein binding region shown in this study.

## INTRODUCTION

The incidence of chronic pneumonia worldwide due to the 2019 coronavirus disease (COVID-19) pandemic that began in late December 2019 is linked to severe acute respiratory syndrome coronavirus 2 (SARS-CoV-2), a novel strain of the genus *Betacoronavirus* in the family *Coronaviridae* (1). The structural makeup of SARS-CoV-2 comprises four structural proteins required for viral assembly in the host cytoplasm, the spike (S), membrane (M), nucleocapsid (N), and envelope (E) proteins, which are encoded by 3′ open reading frames of the viral genome (2, 3). The initiation of pathogenesis is driven by the spike glycoprotein via attachment to the integral receptors on the host cell membrane, such as angiotensin-converting enzyme 2 (ACE-2) and transmembrane protease, serine 2 (TMPRSS2), in humans (4, 5).

The spike protein is a critical determinant of the viral host range and tissue tropism and is a major inducer of host immune responses (2, 6). The S protein of SARS-CoV-2 is a class I viral membrane fusion protein (7, 8) that exists as a trimer, as reported by the cryo-electron microscopy (cryo-EM) structure at a 3.5-Å resolution (2). A total of 22 predicted N-glycosylation sites are found in the spike glycoprotein of SARS-CoV-2 (9). The amino acid sequence of the spike glycoprotein consists of a large ectodomain, a single-pass transmembrane anchor, and a short C-terminal intracellular tail. The ectodomain contains a receptor binding unit (the S1 subunit) and a membrane fusion unit (the S2 subunit) (10). The S2 subunit has two heptad repeat (HR) regions (HR1 and HR2) preceding the transmembrane (TM) domain, a second proteolytic site (S2), and a hydrophobic fusion peptide (11). Electron microscopic imaging illustrated that the spike glycoprotein forms a clove-shaped spike with three S1 heads and a trimeric S2 stalk. Between the S1 and S2 subunits, a furin cleavage site which is unique to SARS-CoV-2 and which is not present in severe acute respiratory syndrome coronavirus (SARS-CoV) is present (12, 13). While the S1 receptor binding domain (RBD) is required to maintain contact with ACE-2, to which it exhibits a high affinity, S2 is the machinery behind the fusion of the two alien membranes and insertion of the viral RNA. Binding of the RBD of S1 to the receptor ACE-2 triggers a series of conformational change in the S2 fusion protein for its transition from a prefusion metastable form to a postfusion stable form, resulting in insertion of the putative fusion peptide into the target cell membrane (33, 34) . This is followed by the association of the HR1 and HR2 domains to form a six-helix bundle fusion core structure, which, in turn, brings the viral envelope and target cell membrane into close proximity for fusion (14, 15).

**FIG 1:**
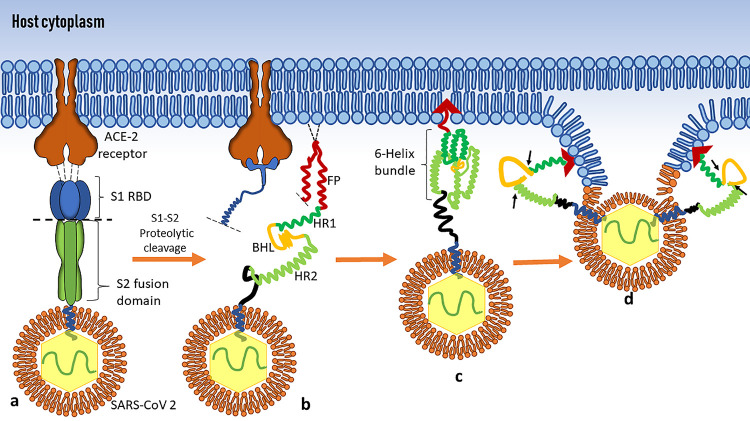
Spike S2-mediated fusion of SARS-CoV-2. (a) The prefusion (trimeric) state of the spike protein recognizing the ACE-2 receptor through the S1 receptor binding domain (RBD) brings a triggering conformational change in the S2 domain, followed by the proteolytic cleavage between the S1 and S2 linker sequences. (b) The energized S2 domain in its prefusion metastable state confronts the host lipid layer via the fusion peptide (FP), which then embeds in the lipid layer due to the abundance of hydrophobic residues. (c) A second conformational change occurs in the heptad repeats (HR1 and HR2), which form a six-helix bundle linked by a beta-hairpin loop, which is crucial for the transition to the postmetastable state, as the beta-hairpin loop acts as a hinge upon end-to-end in-groove attachment of HR1 and HR2 (d). This phenomenon fuses the lipid membranes of the host and virus in an energetically favorable fashion, which is followed by capsid cleavage and RNA insertion downstream.

The precise localization of the fusion peptide (FP) in the S2 fusion protein is indefinite in SARS-CoV-2 and in earlier strains of SARS-CoV. However, various proteomic assays using synthetic peptide replicates have estimated the overlapping sequences upstream of the N terminus of the HR1 domain that correspond to the modulation of virus-host membrane fusion. The fusion peptide sequences of residues 770 to 788 and 816 to 825 and inner FP residues 873 to 888 have been proposed to be the critical portion of the S2 fusion protein determining the fate of membrane fusion in SARS-CoV (15, 16). For SARS-CoV-2, _788_IYKTPPIKDFGGFNFSQIL_806_ was reported to be the important sequence which is involved with membrane fusion (16). The fusion peptide is biochemically characterized by its hydrophobic nature, which is expressed by a higher propensity of nonpolar amino acid residues, like those in glycine (G), alanine (A), phenylalanine (F), and, often, tryptophan (W). This hydrophobic core aids with the host membrane lipid interaction and penetration. A schematic representation of the spike-mediated fusion in SARS-CoV-2 is illustrated in Fig. 1.

The scheme of the present study used a knowledge-based drug repositioning approach, employed against the fusion peptide of the S2 fusion domain of the spike protein in SARS-CoV-2. Using the hydrophobic binding pocket formed from the contributing nonpolar residues, we screened an FDA-approved drug library using molecular docking. To tackle the insufficiency of information on the binding pockets and coordinates, we used an approach similar to that used by Sarma et al. (17). Worldwide, researchers are using their computational and biophysical skills to contribute to obtaining an exhaustive understanding of the machinery and mechanism of SARS-CoV-2, and these efforts have provided certain leads in some studies, some of which have focused on the repositioning of commercially available drugs to achieve a rapid response (18). We have established a computational model, using molecular dynamics (MD) simulations, which describes the stability of these drugs bound to the FP domain. The S protein fusion peptide region has found its gravity in being a vital component of vaccine and monoclonal antibody (MAb) development. However, this is the first study directed toward the identification of viral fusion and entry inhibitors which directly target the FP binding site of SARS-CoV-2 or previous SARS-CoV strains in this regard.

## RESULTS

### Spike S2 fusion domain structure and FP domain binding site.

**FIG 2:**
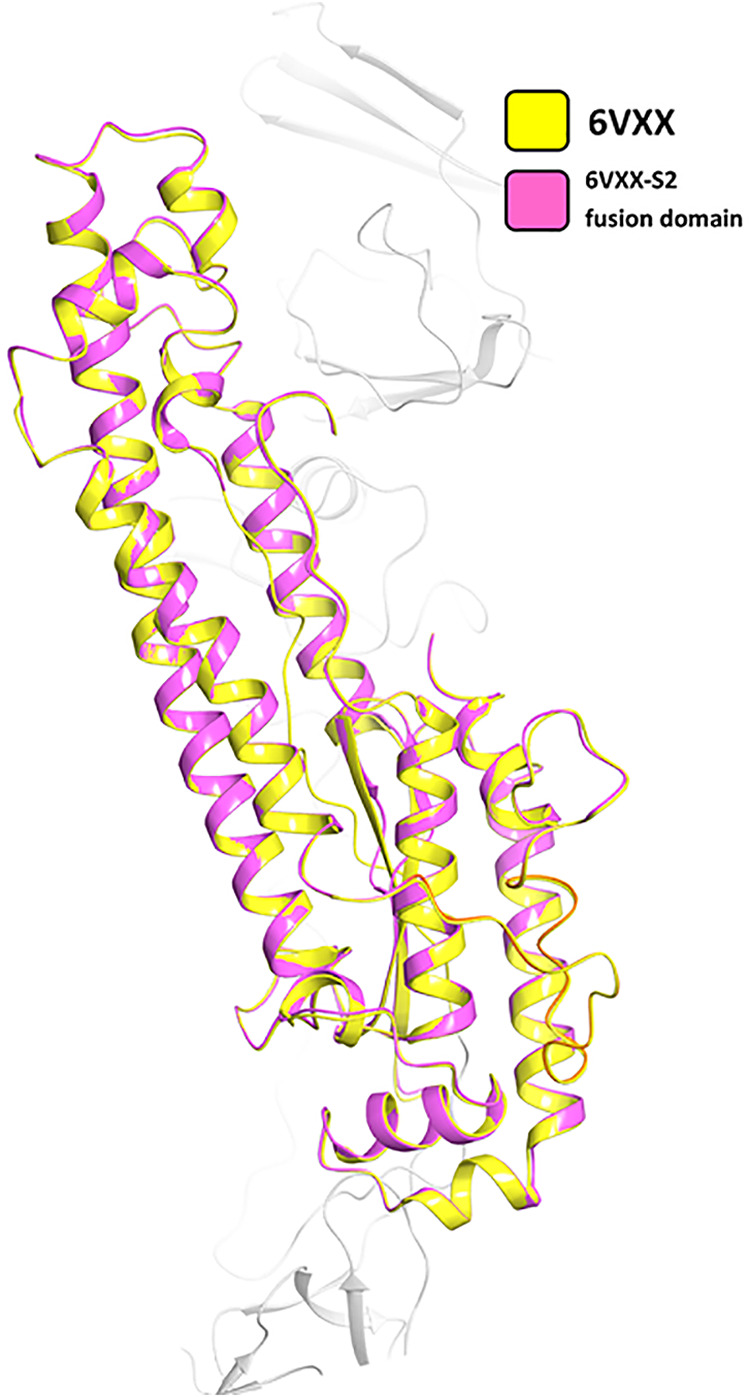
C_α_ superposition of the cryo-EM structure of the SARS-CoV-2 spike glycoprotein (PDB accession number 6VXX) and the S2 fusion domain (S2_fp_) extracted from the structure with PDB accession number 6VXX (RMSD = 0.263 Å).

**FIG 3:**
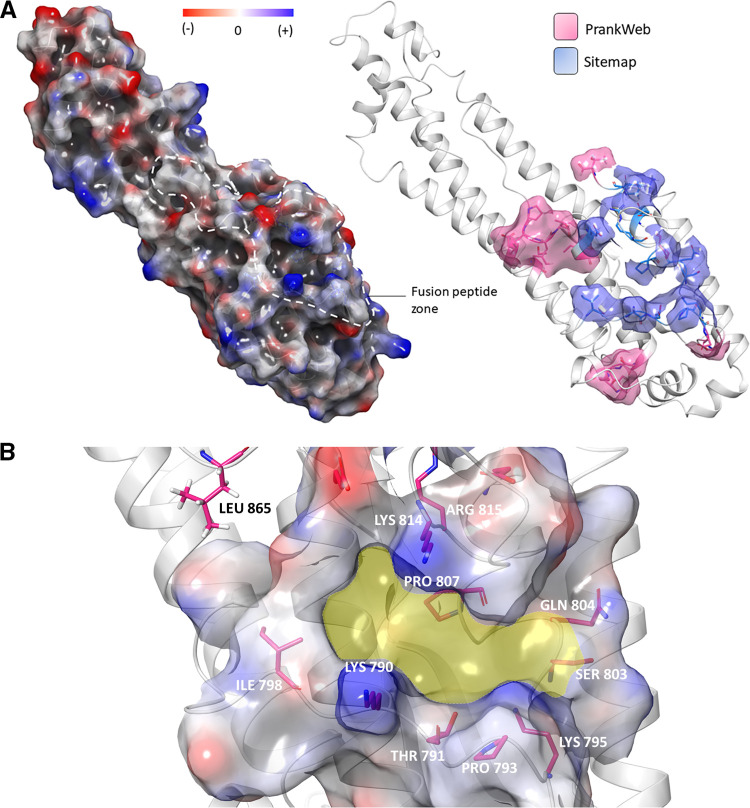
Binding site approximation for the spike S2 FP domain. (A) (Top) The electrostatic potential energy surface diagram features the pockets, and the intensity of the red-blue coloration depicts the polarity of the residues. (Right) The custom representation of the ranked pocket residues is from the PrankWeb and Sitemap web servers. (B) The average pocket localization with a high incidence of docking poses, portrayed by the yellow cavity.

Upon a conclusive comparative array of analyses provided by the PROCHECK program, the structure with PDB accession number 6VXX was carried forward for use in this investigatory study owing to its greater number of residues (2,916 amino acids) and low maximum deviation value (8.1). The extracted spike S2 fusion domain (Ile720 to Gln1071) further used for molecular docking was, contentedly, under a 0.263-Å root mean square deviation (RMSD) upon C_α_ overlay; we called this fragment the S2 fusion peptide-containing domain (S2_fp_) (Fig. 2). The binding pocket features of the residues around the FP region obtained from PrankWeb, a server-based tool, and Sitemap, a Maestro tool, were used to assume an average coordinate between residues Ile770 and Gly880, keeping the previous SARS-CoV FP localization in inclusion (35). The receptor grid for molecular docking was generated amid the residues Asp775, Thr778, Asp796, Gly798, Thr827, Asn856, Thr859, Leu861, Pro863, Thr 866, and Ile870 with reference to the PrankWeb pocket ranks, in addition to Sitemap-estimated residues Ile788, Lys790, Thr791, Pro793, Lys795, Ser803, Glu804, Pro807, Lys814, Arg815, Asp820, Asn824, and Glu868 (Fig. 3A). The generalized binding pocket created by the proposed model is centralized around the residues depicted in Fig. 3B.

### Virtual screening and MD simulations of best-fit FDA-approved drugs.

The only BLAST homology search result for the sequence _788_IYKTPPIKDFGGFNFSQILPDPSKPSKRSF_817_ with a crystallized ligand in the region considered was the protein nitrate reductase of Ulva prolifera (PDB accession number 5YLY), which had 54% sequence identity. A flavin adenine nucleotide (FAD) molecule was crystallized with the homologous Lys, Phe, Tyr, and Ser residues between the matched sequence from residues 679 to 698 via H bonds. The molecular docking (extraprecision [XP] mode) and molecular mechanics/generalized Born model and solvent accessibility (MM/GBSA) calculation of FAD and the principal membrane phospholipids phosphatidyl-l-serine (PS), phosphatidylcholine (PC), and phosphatidylethanolamine (PE) with S2_fp_ was performed (Table 1). Since the binding free energy (Δ*G*_bind_) of protein-ligand complexes gives a better estimate of the binding affinities, these compounds were ranked on the basis of their binding free energies (19). The highest binding energy was −42.266 kcal/mol for PS and, hence, was considered the baseline for the virtual screening that followed.

**TABLE 1:** Binding of knowledge-based reference molecules to estimate minimum binding energy

Molecule name	Docking score	MM/GBSA Δ*G*_bind_ (kcal/mol)	Important residue interactions
Phosphatidylethanolamine	−3.648	−62.39	H bond (Lys795, Ser803), salt bridge (Asp808)
Phosphatidylcholine	−1.231	−51.883	H bond (Lys790, Lys795), salt bridge (Lys790)
FAD	−6.764	−50.823	H bond (Pro807, Lys814, Ile788), salt bridge (Lys814)
Phosphatidyl-l-serine	−4.847	−42.266	H bond (Thr791), salt bridge (Lys790)

The SARS-CoV-2 spike S2 fusion peptide-based virtual screening of FDA-approved drugs delivered a few molecules in their optimal pose at the FP binding pocket, and these were filtered and ranked from those with the highest affinity to those with the lowest affinity for the receptor owing to the MM/GBSA Δ*G*_bind_ values upon the output docked poses obtained with the Glide module (Table 2). Molecules with Δ*G*_bind_ values higher than −42.266 kcal/mol were screened out there and then to obtain the molecules highly attracted to S2_fp_, in contrast to the phospholipids. To our surprise, FAD was also one of the candidates included (Δ*G*_bind_ = −50.823 kcal/mol). These 15 drugs (including FAD) were used for optimization, which was achieved using the results obtained from MD simulation described below.

**TABLE 2:** Result of virtual screen using Glide, showing the docking score and ranked MM/GBSA binding free energy of docked poses^*a*^

Drug name	Docking score	MM/GBSA Δ*G*_bind_ (kcal/mol)
Anidulafungin	−8.774	−88.602
Bleomycin	−9.153	−88.376
Micafungin	−7.743	−67.817
Plicamycin	−6.771	−62.403
Nafarelin	−7.063	−57.569
Edoxaban	−6.834	−57.338
Cefiderocol	−6.936	−56.279
Imidurea	−7.492	−52.435
Chloramphenicol succinate	−6.381	−51.324
FAD	−6.764	−50.823
Imipenem	−5.22	−49.288
Cangrelor	−6.688	−48.58
Arbutin	−6.537	−47.345
Cefonicid	−5.834	−45.845
Fondaparinux	−6.573	−42.516

a Screen result with an energy lower than −42.266 kcal/mol.

**FIG 4:**
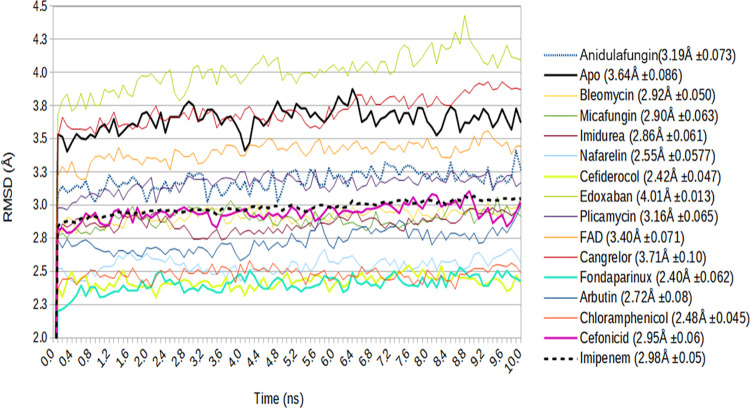
Combined C_α_ RMSD spectra of the fusion peptide (FP) region, representing the averages of the RMSDs at each frame (0.1-ns interval) of the sample trajectories for each drug-protein complex selected after virtual screening, with the reference RMSD line (the black thick line) representing the apo form of the FP region.

**FIG 5:**
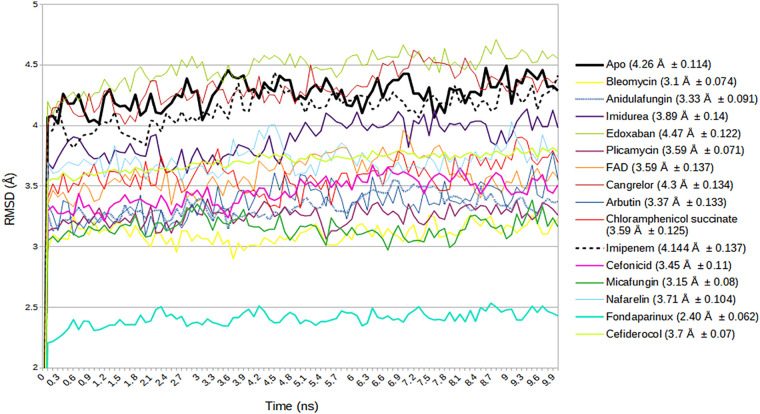
Combined C_α_ RMSD spectra of the S2_fp_ subunit for 10 ns, with each line representing the average RMSD of parallel sample trajectories at each frame (0.1 ns). The black thick line represents the RMSD of the apo form of S2_fp_, a ligand-unbound reference.

**FIG 6:**
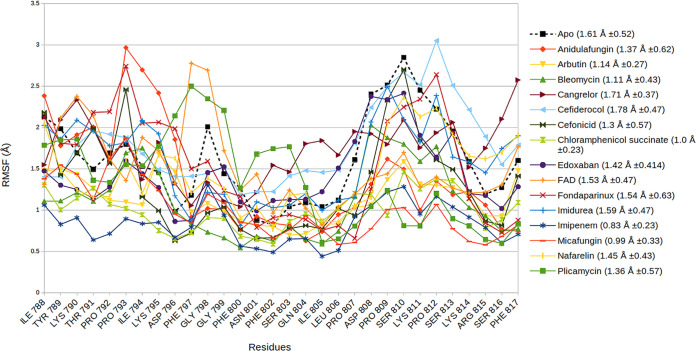
The combined RMSF plot of the C_α_ atom of FP residues illustrates the variation in the fluctuations that comes about when the described drugs occupy the FP binding pocket. The black dotted line represents the RMSF of the apo protein; the average RMSF for the FP residues + standard deviation is provided in parentheses.

Multiple short MD simulations (10 10-ns simulations) were performed for these selected drug molecules, and the results for these 10 sample trajectories were plotted with reference to the results for the apo form. The data obtained for each candidate were the protein (C_α_) RMSD for both the local (FP region) and the global (S2_fp_) domains of the target protein (Fig. 4 and 5, respectively), while the fluctuations of the carbon backbone at the FP region are illustrated by the C_α_ root mean square fluctuations (RMSFs) (Fig. 6) of the drug-protein complexes. These findings, when integrated with the protein-ligand binding profile, provided a much better understanding of the quality of the interaction during the simulations (Table 2).

### MM/GBSA binding free energy of MD trajectories.

**FIG 7:**
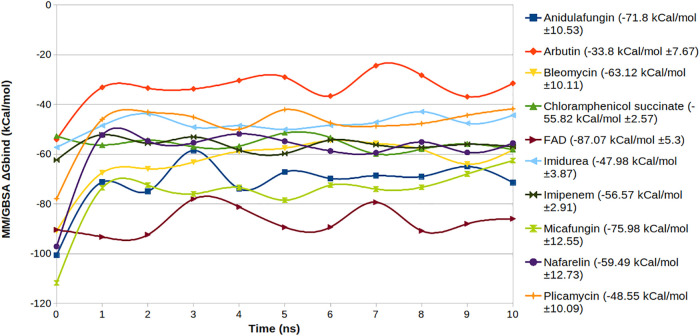
MM/GBSA binding free energy averages for selected drugs with the S2_fp_ protein plotted at 1-ns intervals. The lowest-energy zone is occupied by FAD, while arbutin characterized the highest energy interaction.

The MM/GBSA binding energy estimation of trajectory snapshots was performed for selective candidates that had a good interaction profile and for which the drug-ligand complexes showed structural integrity. The graph in Fig. 7 shows the average Δ*G*_bind_ values at 1-ns intervals for the multiple trajectories. The average overall Δ*G*_bind_, which describes the degree of change in the binding affinity of the drugs with the S2_fp_ protein, with the standard deviation is stated for each molecule.

## DISCUSSION

The molecular docking study gives a faint idea about the binding and slight insights on the chemistry of the interaction. All the selected drugs were chosen on the basis of the hypothesized rationale that the selected molecule must outcompete the lipid constituents which take part in membrane fusion. However, these variables alone are insufficient to explain the relative affinities; hence, the MD simulations were performed. In the results obtained from the MD simulations, it can be seen that the C_α_ RMSD of local FP region and whole S2fp subunit (Fig. 4 and 5) and C_α_ RMSF (Fig. 6) of the fusion peptide fragments (residues Ile788 to Phe817) derive a distinct spectrum of the structural changes encountered from the simulated drugs. The baseline standard for the obtained values is the apo structure (or the structure with no ligand bound), which speaks so much for the positive and negative changes caused by the drug interaction, for which the mean RMSD of all the 10 sample trajectories put together lies at 3.64 ± 0.086 Å. Now, if we are to talk about the comparison of the overall RMSD of the FP region with the apo structure in a view of an ideal drug with a good affinity, we ought to prefer molecules with lower C_α_ deviations than the apo structure, also taking into consideration the standard deviations.

However, this comparison puts away only cangrelor (3.71 ± 0.10 Å) and edoxaban (4.01 ± 0.013 Å) from the picture; hence, the C_α_ RMSF of the FP region adds a dimension to an effective comparison. By comparing the residue fluctuations and the S2_fp_-drug interaction profile (Table 3) with those of the apo structures, we can conclude that the relative stability of the FP region was seen (in increasing overall fluctuations) for imipenem (0.83 ± 0.23 Å), micafungin (0.99 ± 0.33 Å), chloramphenicol succinate (1.0 ± 0.23 Å), bleomycin (1.11 ± 0.43 Å), arbutin (1.14 ± 0.27 Å), plicamycin (1.36 ± 0.57 Å), anidulafungin (1.37 ± 0.62 Å), nafarelin (1.45 ± 0.43 Å), FAD (1.53 ± 0.47 Å), and imidurea (1.59 ± 0.47 Å), whereas cangrelor (1.71 ± 0.37 Å) and cefiderocol (1.78 ± 0.47 Å) were found to have higher FP residue fluctuations than the apo structure (1.61 ± 0.52 Å).

**TABLE 3:** MD interaction profiles of candidate drugs with the SARS-CoV-2 S2 fusion peptide

Compound and residue	Interaction profile	C_α_ RMSF (Å)	C_α_ RMSD (Å)	
			Local	Global
Bleomycin			2.92 ± 0.050	3.1 ± 0.074
Ile788	H bond, hydrophobic	2.12		
Tyr789	H bond	1.9		
Lys790	H bond, hydrophobic	1.7		
Thr791	H bond, water bridges; multiple contacts with highest interaction strength	1.5		
Gln872	H bond; multiple contact points	1.58		
Anidulafungin			3.19 ± 0.073	3.33 ± 0.091
Phe797	Hydrophobic	0.912		
Gly799	Water bridges, H bonds	1.0		
Asn801	H bond and water bridges; excellent, multiple contacts	0.92		
Lys921	H bond, hydrophobic and water bridges; highly attracted to carbonyl group of 10 forms of ionic contacts	2.0		
Micafungin			2.90 ± 0.063	3.15 ± 0.08
Phe762	H bonds and water bridges; strong	1.34		
Lys795	H bonds and strong water bridge, weakly ionic and hydrophobic interactions; triple contacts to ligand subtypes	1.23		
Asp808	Water bridge, good solvent accessibility	0.77		
Gln1005	H bond	1.15		
Imidurea^*a*^			2.86 ± 0.061	3.89 ± 0.14
Thr791	H bond, water bridges, hydrophobic	1.95		
Ile794	H bond, water bridges, hydrophobic	2.08		
Ser803	H bond, water bridges, hydrophobic	1.05		
Asp808	H bond, water bridges, hydrophobic	2.06		
Gln872	H bond, water bridges, hydrophobic	1.38		
Ser875	H bond, water bridges, hydrophobic	1.235		
Leu806	H bonds; strong multiple contacts	1.12		
Pro807	H bonds; strong multiple contacts	1.156		
Nafarelin			2.55 ± 0.0577	2.92 ± 0.050
Thr791	H bonds and water bridges	1.15		
Ile794	H bonds and water bridges	1.2		
Ser803	H bonds and water bridges	1.18		
Pro807	Water bridge and hydrophobic; strong	1.05		
Asp808	H bond and weakly ionic; high strength	1.15		
Pro809	Hydrophobic	2.05		
Lys811	H bond	2.12		
Cefiderocol			2.42 ± 0.047	3.7 ± 0.07
Lys790	H bond and ionic; strong multiple contacts	1.78		
Thr791	H bond and water bridge; strong multiple contacts	1.96		
Lys814	H bond, weakly hydrophobic	2.213		
Glu868	Water bridge; good solvent accessibility	2.05		
Edoxaban			4.01 ± 0.013	2.92 ± 0.122
Thr791	H bond and water bridge; strong	1.7		
Ile794	H bond; weak	1.8		
Leu806	Hydrophobic	2.05		
Plicamycin			3.16 ± 0.065	3.59 ± 0.071
Thr791	H bond and water bridges; adequate	1.36		
Ile794	Hydrophobic; weak	1.53		
Pro807	H bond; weak	0.805		
FAD			3.40 ± 0.071	3.59 ± 0.137
Lys790	H bond, water bridge, and weakly ionic; overall strong multiple contacts	2.37		
Thr791	H bond and water bridges; good overall multiple contacts	2.14		
Lys814	H bond, ionic and hydrophobic; pi cation formation with ligand, high multiple contacts	1.2		
Cangrelor			3.71 ± 0.10	4.3 ± 0.134
Lys790	H bond; adequate	2.33		
Lys795	H bond and hydrophobic interaction; good multiple contacts	1.82		
Fondaparinux^*b*^			2.40 ± 0.062	2.40 ± 0.062
Lys790	H bond, water bridges, and weakly ionic	1.79		
Thr791	H bond, water bridges, and weakly ionic			
Lys795	H bond, water bridges, and weakly ionic; strong multiple contacts	2.05		
Pro807	Water bridges; solvent accessibility	0.65		
Arbutin			2.72 ± 0.08	3.37 ± 0.133
Thr791	H bond and water bridges; multiple contact points	1.25		
Ile794	H bond; weak	1.06		
Pro807	H bond; strong multiple contacts	1.03		
Asp808	H bond and weakly ionic	1.06		
Chloramphenicol succinate			2.48 ± 0.045	3.59 ± 0.125
Thr791	H bond and water bridge; good multiple contact points	1.265		
Lys795	H bond and ionic interaction; good	0.751		
Pro807	H bond, water bridges	0.2		
Imipenem			2.98 ± 0.05	4.14 ± 0.137
Lys790	H bond, water bridge, and ionic; good	0.906		
Thr791	H bond; continuous contact	0.637		
Lys814	H bond, ionic; strong multiple continuous contacts	0.912		
Glu868	H bond, ionic, and water bridges; multiple contacts	1.67		
Cefonicid			2.95 ± 0.06	3.45 ± 0.11
Lys790	H bond, ionic, hydrophobic; adequate	1.7		
Thr791	Water bridge; highly solvent accessible	1.98		
Pro807	H bond; weak	0.93		
Lys814	H bond, ionic, hydrophobic; maximum coverage in simulation interaction	1.22		

a Thr791, Ile794, Ser803, Asp808, Gln872, and Ser875 had equivalent interactions that were extremely confined to the FP region.

b Lys790 and Thr791 had too many nonspecific contacts.

Driving further into the interaction efficiency at the residue level, the fluctuations at Lys790, Leu806, and Pro807 (FP RMSF, 1.42 ± 0.414 Å) upon edoxaban contact were larger than those for the apo structure. Fondaparinux (FP RMSF, 1.54 ± 0.63 Å) showed too many nonspecific contacts and was often found leaving the FP region in most trajectory frames, and even at strong interactions, it had higher residue fluctuations with Lys790, Thr791, and Lys795 than the other drugs tested. The drug cefonicid (FP RMSF, 1.3 ± 0.57 Å) had a weak interaction ratio in comparison to the ratios for the fellow candidate molecules and did not fulfill the interacting residue fluctuation minimization for Lys790 and Thr791. The Spearman rank correlation between the local (FP) and global S2_fp_ RMSD was 0.443; hence, the local and global structural changes in proteins have a low homology in ligand binding iterations.

The MM/GBSA binding free energy data for the selected drugs (imipenem, micafungin, chloramphenicol succinate, bleomycin, arbutin, plicamycin, anidulafungin, nafarelin, FAD, and imidurea) were calculated by exploiting the trajectory frames. Although FAD is a less opportunistic drug candidate and has a history of being withdrawn from treatment trials, testing of FAD paid off by providing and validating the standard average value for the drug binding affinity and providing the profile around which it is more likely to find a probable drug of choice. The binding free energies obtained from the still trajectory drug-ligand poses were plotted for the 10 sample trajectories at random velocities in parallel for 10-ns time series (Fig. 7). The average Δ*G*_bind_ values and their standard deviations facilitate a fine characterization of the bound drugs at the FP pocket. The degree of variation in the free energy trendlines speaks to the steadiness of the drug-ligand binding of the S2 domain overall.

Recalling that the Δ*G*_bind_ maximum was set at −42.266 kcal/mol, it can be concluded with convenience that arbutin (−33.8 ± 7.67 kcal/mol) expresses a lower affinity for S2_fp_ than the other compounds tested. Moreover, micafungin (−75.98 ± 12.55 kcal/mol), anidulafungin (−71.8 ± 10.53 kcal/mol), bleomycin (−63.12 ± 10.11 kcal/mol), plicamycin (−48.55 ± 10.09 kcal/mol), and nafarelin (−59.49 ± 12.73 kcal/mol) underwent higher energy fluctuations in the first few nanoseconds than the other compounds tested, but their stability for the last 3/4 of the simulation cannot be overlooked, and with their extremely low Δ*G*_bind_ values, they manifested an excellent affinity for S2_fp_; still, the standard deviations speak for the drug-protein binding itself. Significantly stable and responsive energy trends can evidently be observed in the binding energy timeline of chloramphenicol succinate (−55.82 ± 2.57 kcal/mol), imipenem (−56.57 ± 2.91 kcal/mol), and imidurea (−47.98 ± 3.87 kcal/mol). These low fluctuations can be attributed to the least interrupted binding of these molecules with the fusion peptide residues. The remarkably lowest energy of FAD with quite a low deviation (−87.07 ± 5.3 kcal/mol) has proven to be fortuitous evidence that we did not look for. The energy trend for FAD (Fig. 7) shows that it has an engrossingly high affinity for the FP pocket. Just as in the case of the Ulva prolifera nitrate reductase protein, perhaps a shared homology in the sequence of the binding cleft might also allow us to speculate about the homology of the affinity.

**FIG 8:**
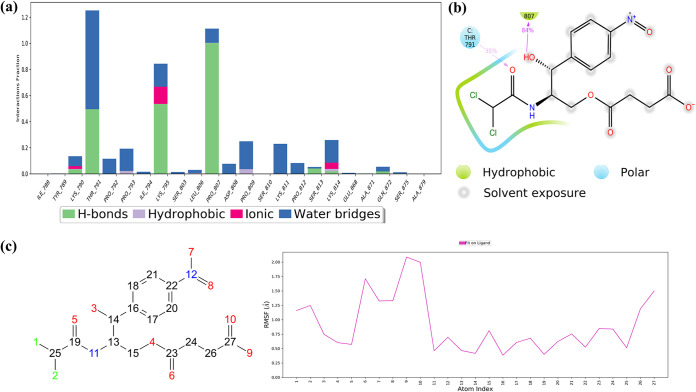
Ligand interaction properties of chloramphenicol succinate, showing its interaction ratio (a) and interaction strength (b) with S2_fp_ residues. (c) Atomic fluctuations, which represent the relative activity of the functional groups with respect to the initial conformation.

Regardless, chloramphenicol succinate (the prodrug of chloramphenicol), a bacteriostatic antibiotic often used to treat chronic bacterial infections as a plan B drug, indicated excellent interactions and potent interactions in MD simulations. This balanced reactivity of chloramphenicol succinate can be accredited to its evenly dispersed carbonyl and hydroxyl groups, which provides maximum solvent exposure to facilitate water bridges and H bond formations with the important FP residues (Thr791, Lys795, Pro807, and Lys814) (Fig. 8a and c). Chlorine atoms form halogen bridges (halogen-water-hydrogen) with hydrophobic Pro793, which provides a bonus anchorage to the FP region (Fig. 8b). The average displacements of residues were as low as 1.0 ± 0.23 Å (FP RMSF) and 2.48 ± 0.045 Å (FP RMSD); still, the global S2_fp_ RMSD grew to a value of up to 3.6 ± 0.23 Å, which, then again, was lower than that of the apo form. The substantially steady Δ*G*_bind_ value further adds to its positive interaction with S2_fp_.

**FIG 9:**
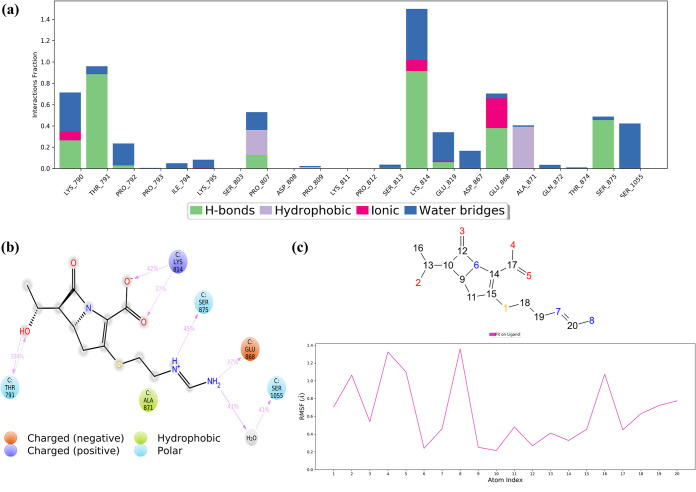
Ligand interaction properties of imipenem, showing its interaction ratio (a) and interaction strength (b) with S2_fp_ residues. (c) Atomic fluctuations of imipenem elements.

Imipenem, a semisynthetic derivative of thienamycin, is used to treat bacterial infections often associated with the respiratory, female reproductive, and urinary tracts. In comparison with the other drugs tested, it exhibited the most minimized residue fluctuation and a rich multiple-contact profile, with higher H bond percentages with carboxyl, hydroxyl, and amino groups. These characteristics maintain a steady conformation of the imipenem molecule upon interaction with FP residues (Lys790, Thr791, Pro807, Lys814). This finding was also supported by additional interactions with certain residues downstream of FP which share a close proximity at the fusion peptide region (Ala871, Glu868, Ser875, and Ser1055) (Fig. 9a and b). Perhaps this multidirectional anchorage effect keeps the FP residues from fluctuating higher, i.e., an FP RMSF of 0.83 ± 0.23 Å. For the atomic-level fluctuation that takes place during the simulation, see Fig. 9c. The physical stability of the FP region is further validated by the FP RMSD, which was equal to 2.98 ± 0.05 Å, but then again, it reached a global RMSD maximum of 4.14 ± 0.13, which differed very slightly from that of the apo form (4.26 ± 0.11 Å). However, from the MM/GBSA of imipenem’s trajectory, it can be inferred that its interaction has a significant overall minimization of the S2_fp_-imipenem complex.

**FIG 10:**
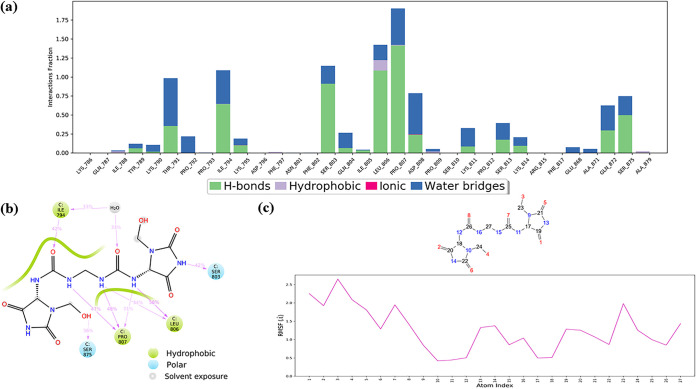
Ligand interaction properties of imidurea, showing its interaction ratio (a) and interaction strength (b) with S2_fp_ residues. (c) Atomic fluctuations of imidurea atomic elements.

Lastly, imidurea (or imidazolidinyl urea), an antimicrobial preservative, is characterized by a maximum number of contacts specific to the FP region, and that, too, occurs with high residue interaction percentages (Fig. 10). The equally dispersed amino-carbonyl ratio in the imidurea molecule makes it an H bond magnet for the surrounding residues of most FP region residues. The middle amino-carbonyl chain binds FP at buried sites, where it is encircled by hydrophobic amino acids. The relative contribution of FP residues in stabilizing the overall S2_fp_ structure is the highest for imidurea. Imidurea, too, has structural integrity maintained with highly convincing attributes, i.e., an FP RMSF of 1.59 ± 0.47 Å, an FP RMSD of 2.86 ± 0.06 Å, and a global S2_fp_ RMSD of 3.89 ± 0.14 Å, and this structural integrity is thoroughly supported by a steady Δ*G*_bind_ value.

**FIG 11:**
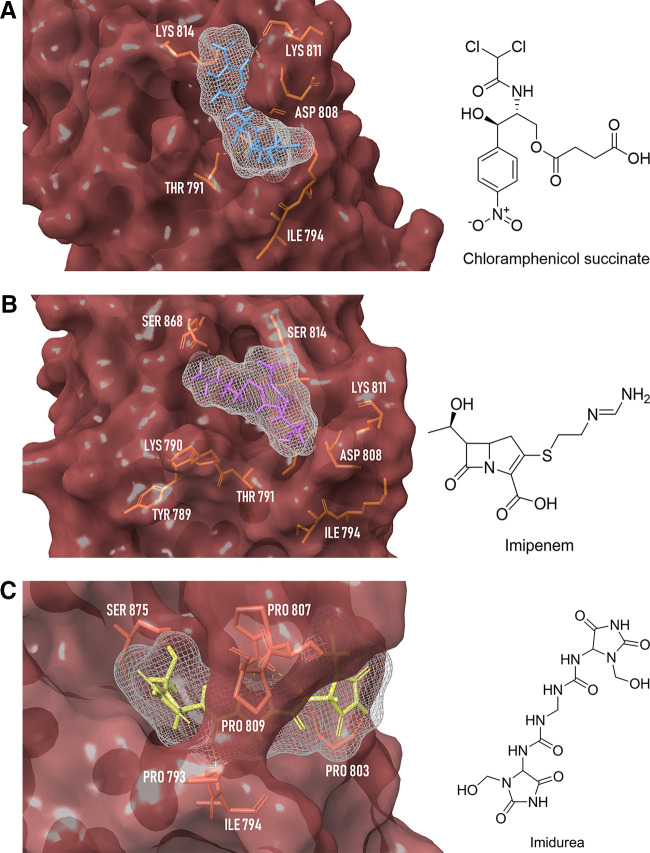
Surface diagram of the SARS-CoV-2 spike S2 fusion peptide region bound with chloramphenicol succinate (A), imipenem (B), and imidurea (C). Yellow dotted lines, H bond; pink, salt bridge.

The binding poses of these three drugs in one of the last few stable trajectory frames with the fusion peptide reveal a very important feature about the FP region. The FP region of SARS-CoV-2 is highly flexible and has a substantially unstable topology; the same can be seen in Fig. 11A to C. Note that the respective conformation of the same amino acids is highly different owing to the stable conformation adapted by each drug molecule. In the case of imidurea, a hole in which imidurea is embedded is formed (Fig. 11C). These drug molecules reduce the reactivity of the fusion peptide residues, which results in resistance in the overall fluctuation of the large reactive loop forming between Ile788 and Phe817. In conclusion, these selected drugs, chloramphenicol succinate, imidurea, and imipenem, fulfilled several criteria that identified them as probable ligands specific for binding to residues of the FP region on computational grounds that bring receptor-based docking, MD simulations, and binding free energy of complexes together as a protocol. Analysis of these drugs may be advanced to exploratory studies that may validate their role or perhaps their efficacy at inhibiting the virus-host fusion machinery of SARS-CoV-2.

### Conclusion.

The *in silico* drug repositioning study that was conducted and that is described here was inspired by the deadly COVID-19 pandemic occurring around the globe. A virtual screening using molecular docking, MD simulations, and free energy calculation-based methods was employed for the selection of viable experimental molecules from among FDA-approved drugs. Chloramphenicol succinate, imipenem, and imidurea were observed to have the finest interaction profile with FP upon detailed sampling of trajectories from MD simulations run simulated under near physiological conditions (temperature = 310 K, pH 7.4, NaCl concentration = 0.15 mol, pressure = 1.01325 bar). The computational attributes produced in this study provide a viewpoint and computed estimate that the described drugs qualify to be potent inhibitors of SARS-CoV-2 upon optimal contact with the defined fusion peptide (FP) region of the SARS-CoV-2 spike S2 fusion domain. Hence, these molecules could be considered in the development of novel therapeutic alternatives to counter COVID-19 and in experimental assessments of agents that may be used to counter COVID-19. Moreover, the fusion peptide region may find greater importance if not in small-molecule discoveries then in the discovery of peptide inhibitors, a vaccine epitope, or synthetic molecules. The in-depth understanding of the fusion machinery as a functional domain may provide an opportunity to terminate the viral infection prior to viral intrusion into the host.

## MATERIALS AND METHODS

### Computing system.

The following work flows were performed on a Dell Precision Tower 3630 workstation (with 32 GB of random-access memory and an Nvidia Quadro RTX 4000 central processing unit [8 GB]) with an Ubuntu 18.04 LTS Linux distro operating system.

### Protein and ligand preparation.

To obtain the crystal structure of the spike protein of SARS-CoV-2 with 100% sequence identity, the FASTA sequence for GenBank accession number QIC53213.1 was used as the query sequence in an advanced search of the Protein Data Bank (PDB). Comparisons of the scores provided by the PROCHECK program for the structures with PDB accession numbers 6VXX, 6VYB, and 6VSB were used to select the optimal spike protein structure (20). Docking state protein preparation was carried out with the PrepWizard tool in the Maestro program from Schrödinger, LLC (21). Missing side chains and loops were filled, and H bonds were optimized and minimized at physiological pH on the OPLS3 (Optimized Potentials for Liquid Simulations version 3.0) force, extracting one monomeric chain out of the trimer. The S2 domain portion of the protein was extracted to create a new protein entry from Val736 to Gln1071 to sustain the stability within the confined region and was again minimized and superimposed with the native structure with PDB accession number 6VXX for protein structure validation. To assess the binding of FDA-approved drugs with the fusion peptide, a mixed rationale of binding site prediction was employed using the sequence range of previous SARS-CoV FP sequence positions, PrankWeb scores, and the Sitemap web server (22, 23). Receptor coordinates for structure-guided docking were created at the mean position for all the top-scoring pocket residues from PrankWeb and Sitemap. A total of 2,625 approved small molecules from DrugBank was retrieved and three-dimensionally prepared with the LigPrep program (release S.3, 2016; Schrödinger, LLC, New York, NY) with a maximum of up to 32 conformers for each drug under the OPLS3 force field.

### Virtual screening and binding energy calculations.

On the basis of the fusion machinery and the relevant interaction of the fusion peptide leading to the initiation of virus-host membrane fusion, analysis of the interactions of different phospholipid units was studied using receptor-based docking at the estimated binding site in the fusion peptide region. This analysis provided insight into the nature of the interaction between the host lipid layer and the fusion peptide, reflected through Δ*G*_bind_, the interaction profile, and the active residues, depicting the relative affinities for the lipid components. This computational estimate of membrane phospholipid unit interaction provided a graphic simulation of the concept behind the work of Guillén et al. (24). The second approach to search for active molecules with an affinity to bind FP was a site-dependent BLAST search of the FP fragment in the PDB database to detect closely related sequences and, therefore, motifs with a conjugated ligand or any small molecule. These operations were led by the assumption that there must be a minimum binding threshold beyond which the FP is attracted to a foreign molecule.

These methods provided this study with a virtual standard to align the screening outputs to obtain optimal leads on the FP binding pocket. The module Glide was used for tandem screening of the FDA-approved drugs via high-throughput and exhaustive mode molecular docking (high-throughput virtual screening [HTVS], standard precision [SP], and extraprecision [XP] modes) (25). Enhanced sampling for exhaustive flexible docking in the XP mode with a minimum of 3 docking states was used for each molecule. Elicitation of the endpoint binding free energy was done using MM/GBSA in the Prime module (Schrödinger, LLC, New York, NY, 2019). The implicit model VSGB with the OPLS3 force field was selected, and the protein flexibility degree of freedom was confined within 5.0 Å.

### Molecular dynamics simulation.

Molecular dynamics simulations are routinely used to investigate and infer the molecular-level mechanism for microbial pathogenesis but are always open for improvements in the algorithmic accuracy and analytical approaches that might bring out the best estimate of the biological subsystem of subjects. To optimize the estimates of the affinities of binding of the receptor-based screened molecules to the S2 fusion peptide obtained, 10 10-ns sample MD simulations were run for each molecule.

MD simulations for selected spike S2-drug complexes were performed with the Desmond program from D. E. Shaw Research (DESRES) (26). A periodic boundary cuboidal box of 10 by 10 by 20 Å was filled with TIP3P water models, minimized S2-drug complexes, counterions, and salt solution at pH 7.4. The 10 10-ns simulation was programmed at the NPT ensemble class with a Nose-Hoover thermostat and Martyna-Tobias-Klein barostat at 310 K to mimic the physiological temperature on randomized velocities, where the number of molecules (*N*) was roughly between 40,100 and 40,200 (27, 28). Trajectory frame data, including protein RMSD and RMSF and the protein-ligand interaction profile, were obtained for both the global and the local (FP binding pocket) region of the protein with the aid of the Desmond, VMD, and R programs (29–31).

### Calculation of binding free energy of trajectory frames.

The MM/GBSA binding free energy (Δ*G*_bind_) was calculated using the drug-protein trajectory coordinates at equal intervals of 1 ns and the structures obtained from the trajectory snapshot window in Desmond software. To track the generalized trendline of the binding free energy, each interval was made to be 1/10 of the total frames (*n* = 100). MM/GBSA snapshots for each trajectory were individually calculated using Prime software, which defines the binding energies (PE) in the algorithm as Δ*G*_bind_ = PE_complex_ − PE_free ligand_ − PE_free protein_ (32).
